# Proceedings of the world summit on parkinson’s disease

**DOI:** 10.1038/s41531-025-01123-8

**Published:** 2025-10-14

**Authors:** Sneha Mantri, M. Felice Ghilardi, Nicole Lessard, Monica Norcini, Alessandro Di Rocco, Kristin Wallock, John Lehr, Michael S. Okun

**Affiliations:** 1 Parkinson’s Foundation, New York, NY USA; 2 Duke University School of Medicine, Durham, NC USA; 3 CUNY School of Medicine, New York, NY USA; 4 Fresco Parkinson Institute Italia Onlus, Fiesole, IT Italy; 5 Northwell Health, New York, NY USA; 6 University of Florida, Fixel Institute for Neurological Diseases, Gainesville, FL USA

**Keywords:** Diseases, Health care, Neurology, Neuroscience

## Abstract

This meeting report summarizes the proceedings of the inaugural World Summit on Parkinson’s Disease, held 11-13 June 2025 in Fiesole, Italy. The Summit was co-sponsored by the Parkinson’s Foundation and the Fresco Parkinson Institute. Representatives from movement disorders centers, non-governmental organizations, and disease foundations came together to outline key global priority areas for policy-makers, clinicians, and people with Parkinson’s disease.

## Introduction

Parkinson’s disease (PD) is the fastest growing neurodegenerative disease worldwide^1^, with an estimated prevalence of over 25 million by 2050^2^ and the greatest projected increase in areas with lower socio-demographic indices (greatest social deprivation)^3,4^. Recognizing the global impact of the disease, the Parkinson’s Foundation and the Fresco Parkinson Institute convened a steering committee of experts to inform and to initiate planning for a large World Summit on Parkinson’s Disease.

The inaugural meeting was held in Fiesole, IT, in June 2025 and was focused on a global approach to Parkinson’s disease care, exploring what people with PD, their care partners, and clinicians will need across different regions, and how multi-national organizations can better support people with PD today and into the future. Participants identified global PD needs with the highest impact through collaboration of various organizations.

## Meeting Summary

**Fig. 1:**
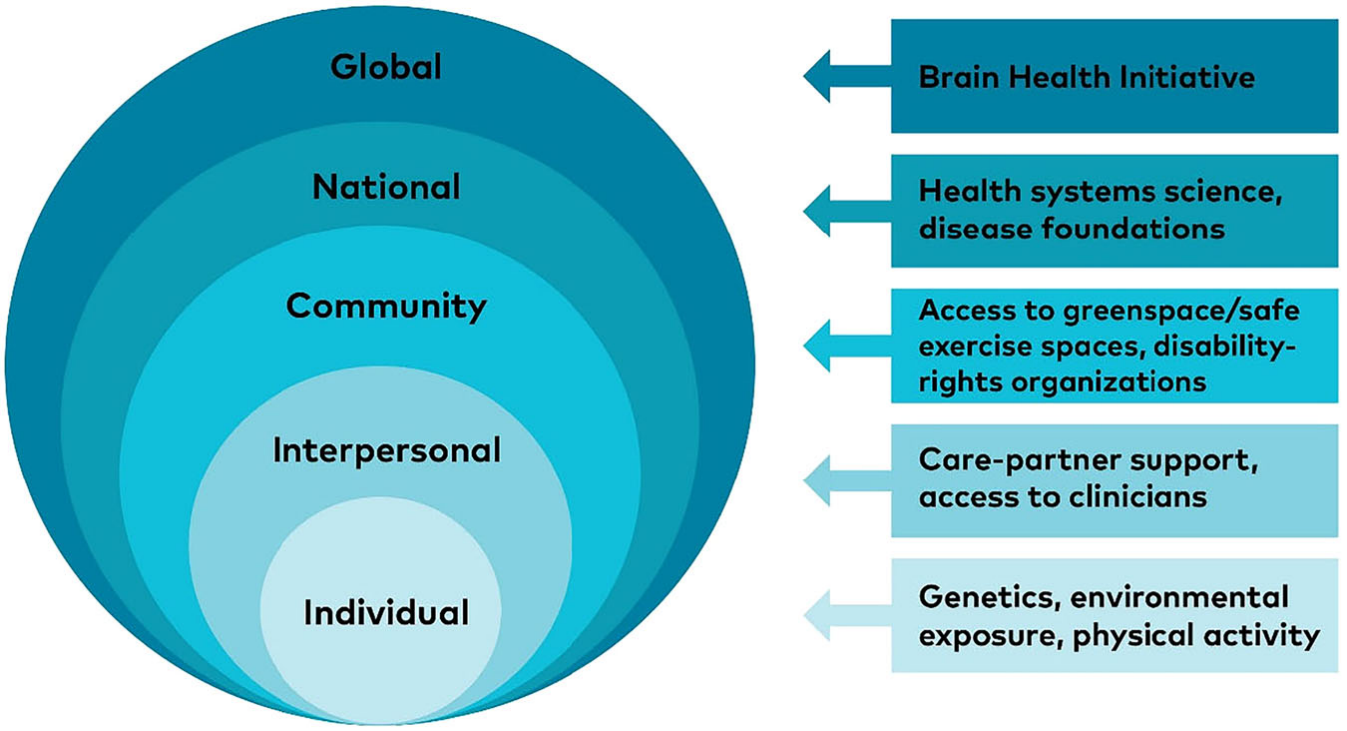
Socioecological Model of PD.

The meeting participants included 26 attendees representing 11 countries across five continents and six international health-related organizations and foundations (Table 1). The meeting format consisted of two group dinners with open discussion and networking, plus 1.5 days of facilitated breakout group discussion (Table 2). There was reporting and synthesis of key themes, takeaways and next steps. The socioecological model of disease (Fig. 1) was used to inform development of the key discussion questions.

**Table 1:** Meeting Participants and Affiliations

Participant	Affiliation	City/Country	Continent
Paolo Calabresi, MD	Fondazione IRCCS Policlinico Gemelli Catholic University	Rome, Italy	Europe
Max Coslov, MPhil	Edmund J. Safra Foundation	Geneva, Switzerland	Europe
Silke Cresswell, MD	University of British Columbia	Vancouver, British Columbia, Canada	North America
Alessandro Di Rocco, MD	Northwell Health; Fresco and Parkinson’s Foundation Board	New York, New York, USA	North America
Rachel Dolhun, MD DipABLM	Michael J. Fox Foundation for Parkinson’s Research	New York, New York, USA	North America
Tarun Dua, MD	World Health Organization	Geneva, Switzerland	Europe
Felice (Lice) Ghilardi, MD	Fresco Parkinson Institute Italia	New York, New York, USA	North America
Ihtsham Haq, MD FAAN	University of Miami	Miami, Florida, USA	North America
John Lehr, MA	Parkinson’s Foundation (President and CEO)	Washington, DC, USA	North America
Nicole Lessard, RN	Parkinson’s Foundation (Chief Clinical Affairs Officer)	Atlanta, Georgia, USA	North America
Sneha Mantri, MD MS	Duke University/Parkinson’s Foundation Chief Medical Officer	Durham, North Carolina, USA	North America
Danni Manzi,	Parkinson’s UK	London, England, UK	Europe
Janis Miyasaki, MD MEd FRCPC FAAN	University of Alberta	Edmonton, Alberta, Canada	North America
Cathy Molohan, DPhil	Parkinson’s Advocate	Frankfurt, Germany	Europe
Elena Moro, MD	European Academy of Neurology	Lyon, France	Europe
Monica Norcini, PharmD PhD	Fresco Parkinson Institute Italia	Florence, Italy	Europe
Njideka Okubadejo, MBCHB FMCP	University of Lagos	Lagos, Nigeria	Africa
Michael S. Okun, MD	University of Florida	Gainesville, Florida, USA	North America
Eli Pollard, MA	World Parkinson’s Coalition	Brooklyn, New York, USA	North America
Artur Schuh, MD PhD	Universidade Federal do Rio Grande do Sul	Porto Alegre, Brazil	South America
Michael Schwarzchild, MD PhD	Massachusetts General Hospital/Harvard University	Boston, Massachusetts, USA	North America
Frederic Seghers, MS	Clinton Health Access Initiative	Ghent, Belgium	Europe
Cholpon Shambetova, MD	Kyrgyz State Medical Academy, University of Luebeck	Bishkek, Kyrgyzstan	Asia
Caroline Tanner, MD PhD	University of California, San Francisco	San Francisco, California, USA	North America
Kristin Wallock, OTD MS OTR/L	Parkinson’s Foundation	Stillwater, Minnesota, USA	North America
Benjamin Walter, MD MBA	Cleveland Clinic	Cleveland, Ohio, USA	North America

**Table 2:** Breakout discussion questions

Session	Questions
Session 1: Epidemiological and Economic Impacts of Parkinson’s Disease	Worldwide epidemiology of PD: What is the situation in your country, region, or part of the world?
	What are the costs associated with PD in your country or region, for patients and for society? (to include the costs of labor – direct and indirect). Considering the cost of PD care in your region, is there a baseline standard of care that is sustainable given your healthcare system?
	What proactive measures or interventions are being taken in your country that help prevent or “adjust the curve” in PD incidence and mitigate progression?
Session 2: Parkinson’s Care Landscape	What does the current care journey look like for individuals living with Parkinson’s disease in your country, region, or local context—from diagnosis through advanced stages? Consider factors such as cultural norms, access to care, systemic barriers, care settings, and the roles of different providers.
	Is it feasible to have general principles of PD care that are valid across settings worldwide?
	The caregiver: burden, support, wellness, necessities, voids…what is the current reality in your country/region? What is achievable for the future?
Session 3: Personalized Medicine and the Impact of AI on the Future of PD Care	The future is here: what are the possibilities and opportunities with AI? Can AI be a tool to spread better care of PD in remote places, a way to increase access, etc.?
	Who are the key partners that could help bring possibilities to fruition? Should the PD community engage with the AI industry like current collaborations with the pharma?
	How is research, including AI research, impacting global care? (earlier diagnosis, prevention, etc.)

The opening session, presented by summit facilitator Michael Okun, MD, focused on key insights on PD trends, prevalence, research, care, and future outlook. Dr. Okun highlighted the rapid rise in PD prevalence worldwide^1^, as well as the marked disparities in access to care^3^, and this was particularly notable in low-middle income countries (LMIC), but also present regionally in high income countries^5,6^. Additionally, Dr. Okun framed the changing PD care landscape, including the development of integrated care models^7^ and new technologies for both monitoring symptoms and providing guidance to people with PD^8,9^. Following this, participants divided into regionally-organized breakout groups (North America, Eurasia/Africa, Foundations/Organizations) to discuss focus areas and to create detailed reports.

## Focus areas and key takeaways

### Epidemiological and economic impacts of PD

While most epidemiological studies are undertaken in high-income countries, all three breakout groups agreed on the increase in PD across their respective regions. A variety of factors contribute to this increasing global prevalence, including an aging population, increased life expectancy, as well as exposure to an increasingly toxic environment (e.g. widespread use of agricultural pesticides, microplastics, heavy metals, contaminated water, and other environmental pollutants)^10,11^. The use of these toxicants varies worldwide, based on regulations—or lack thereof—by national governments. However, because toxicants are spread to distant sites through air and water^12^, people who are not directly exposed to the chemical (e.g. through occupational or recreational use) may still suffer negative neurological consequences.

**Fig. 2:**
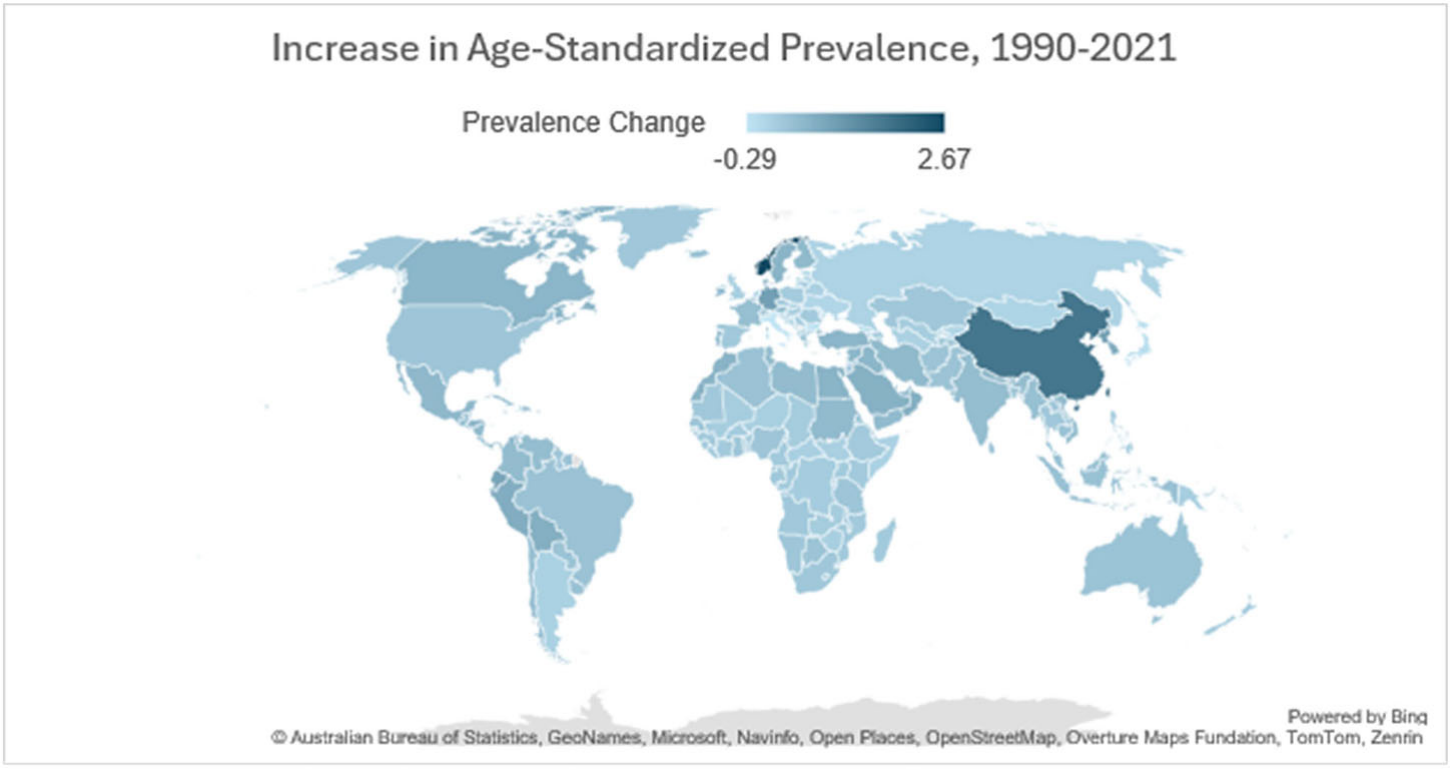
Percent increase in prevalence of Parkinson’s disease, 1990–2021. Data derived from the Global Burden of Disease Visualization Hub (Institute for Health Metrics and Evaluation (IHME). GBD Results. Seattle, WA: IHME, University of Washington, 2024. Available from https://vizhub.healthdata.org/gbd-results/. (Accessed 28 JUL 2025).

High-income countries have also observed an improvement in the diagnostic certainty of PD, in part due to the development of advanced diagnostic technology (e.g. DaTscan, skin biopsy, acknowledgement of the prodromal signs of PD)^13^. By contrast, in low-resource settings, PD remains under-diagnosed and under-treated^14^, in part due to lack of public awareness, lack of trust in medical personnel, and lack of access to clinicians trained to recognize and to address PD signs and symptoms^15^. Nevertheless, LMIC are likely to bear the greatest percent change in PD incidence and prevalence over the next 25 years, with prevalence in some regions more than tripling as a result of population growth and ageing (Fig. 2).

**Fig. 3:**
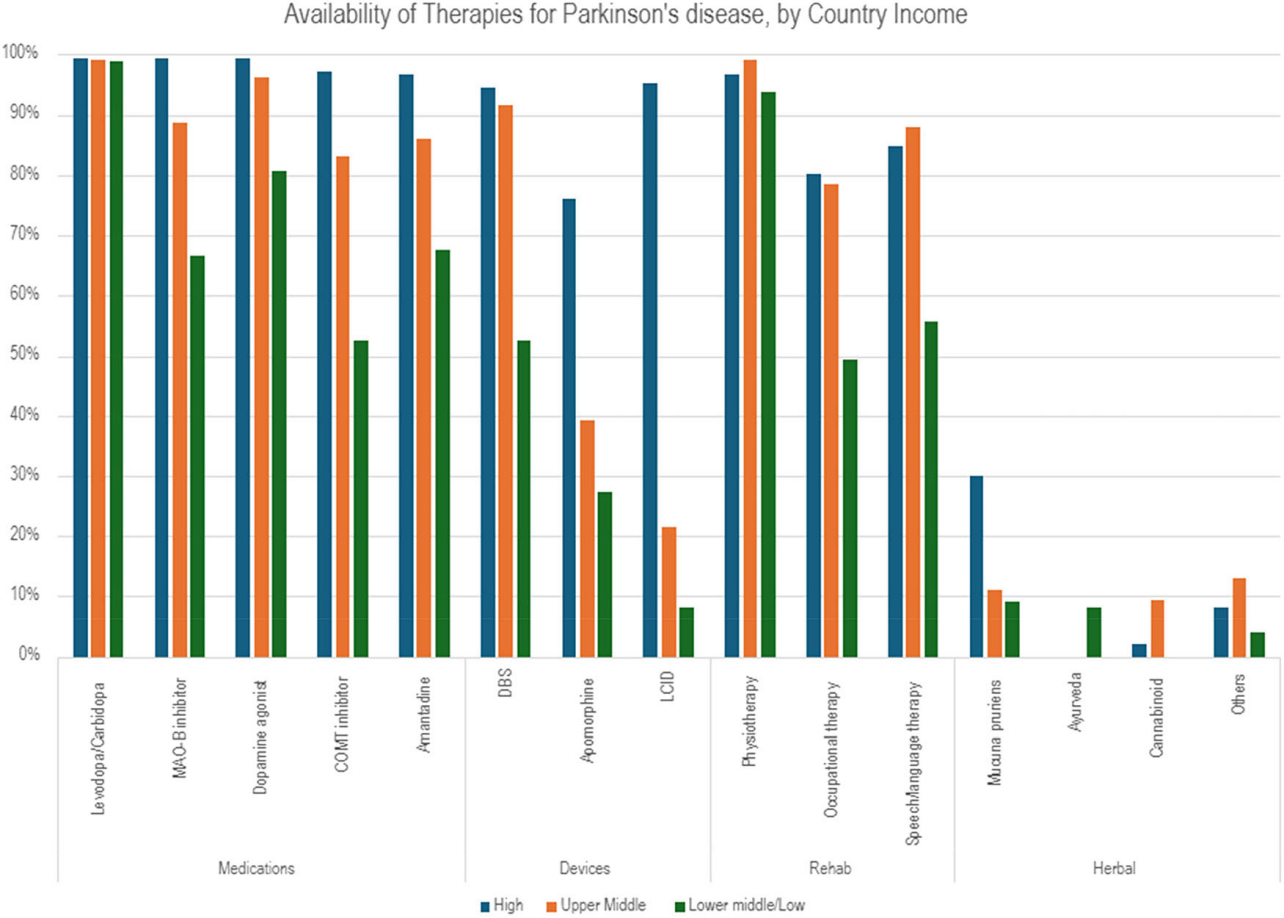
Availability of Therapies for Parkinson’s Disease, by Country Income. Data derived from Goh et al. J Parkinsons Dis. 2022;12(3):1023-1034. 10.3233/JPD-213006.

Similarly, the economic burden of PD varies across the globe. In many LMIC, out-of-pocket costs for basic PD care may exceed the average monthly wage^15,16^, and levodopa and other therapies may not be available in pharmacies or on drug formularies (Fig. 3). Combined with the likely loss of income for those afflicted by PD, these circumstances can lead to catastrophic impacts at the individual, family, and societal levels^17^. Even in relatively well-resourced countries, inequities persist along axes of rurality, socioeconomic status, and education level^18,19^. For instance, in New York City, patients receiving care at a public hospital had higher rates of health care utilization and lower rates of physical therapy referrals, compared to patients receiving care at a private academic hospital a few blocks away^20^. These disparities are most marked at advanced disease stages, which often require expensive, poorly reimbursed therapies. This circumstance is partially the result of for-profit care systems disincentivizing quality care for chronic illnesses such as PD^21^, further worsening a downward care spiral.

The steering committee recommends that epidemiological data gaps be closed immediately via national registries and international collaborations. Among the proactive measures suggested during the discussion there were strategies to increase the awareness of the disease, to build trust in the communities for early intervention and care, and to address the worldwide lack of specialists in movement disorders. Further, the economic argument in favor of early diagnosis and comprehensive, pre-habilitative care should be prioritized at the country/regional level to maximize quality of life and to minimize disease burden.

### Parkinson’s global care landscape

High-quality care for people with PD consists of (1) lifestyle improvements including diet and exercise; (2) access to physical symptom reduction through the use of dopamine replacement therapy; (3) access to mental health support to reduce depression, anxiety, and other factors impacting quality of life for both patients and care partners. Nevertheless, access barriers persist globally for people with PD, driven by both increasing demand (e.g. increased prevalence, increased complexity) and decreasing supply (e.g. lack of trained specialists in PD care). In the United States, for instance, there are fewer than 700 movement disorders specialists for more than one million people living with PD^22^, leading to long waitlists, diagnostic delays, and inappropriately long wait times between visits^23^. In many LMIC countries, there may be fewer than one neurologist of any type per 100,000 people^24^. Furthermore, basic PD treatments, including levodopa, physical therapy, and dietary modifications, may be difficult or impossible to obtain in some regions (Fig. 3)^14,25^, further compounding the disease-associated disability and care partner strain^26^. Care partners of people with PD provide a significant amount of uncompensated care, often at the detriment of their own careers and health, and health systems in both high-income and LMIC regions rarely account for the needs of the carers^27^.

In response to both diagnostic and treatment delays, as well as the scarcity of expert neurologists, some countries have developed community health worker training programs^28^, in an effort to increase front-line capacity. Others have developed hub-and-spoke networks^29^ or primary care nursing integrations^30^ to expand access to high-quality care. Nevertheless, these models require ongoing investment by regional and national health services, as well as regular training of the clinicians themselves to remain up-to-date with best practices in PD care.

The steering committee made a number of strategic recommendations, including the development of training modules for primary care and community health workers. The implementation of regional, or even international, hub-and-spoke care models to address some of the fragmentation of care seen in both LMIC and high-income countries. Alternatively, the development of PD-specific toolkits, akin to the epilepsy toolkits designed by the World Health Organization^31^, may help in an effort to disseminate practice guidelines to community-based clinicians in real time. Innovative models such as the Living Classroom model^32^, can aid in developing specialized long-term care solutions for people with PD, benefiting both the person with the disease and their care partner(s). Lastly, evidence-based, policy-linked minimum care standards can provide a benchmark for countries to measure themselves. For instance, it was clearly stated that one of the first approaches in LMIC should be to make levodopa available through local production, rather than reliance on donations.

### AI, digital health, and personalized medicine

The technological advances of the first quarter of the 21^st^ century, including the rapid uptake of artificial intelligence (AI) in health systems, present both promises and risks to the care of people with PD^9^. Key opportunities include the potential for passive monitoring of symptoms with wearable devices^33^ and even remote diagnosis through facial recognition technology^34,35^ or handwriting analysis^36^. Additionally, AI-driven triage and decision support tools can improve non-specialists’ confidence in making treatment recommendations and rendering appropriate referrals^37,38^, thus potentially mitigating the lack of access to movement disorders specialists. Lastly, predictive models, combining genetics, biomarkers, environmental exposure, and subclinical symptomatology, can aid the early detection of PD at the stages where disease-modifying therapies could have their greatest impact^39,40^.

Nevertheless, significant ethical concerns remain regarding the widespread use of AI and digital health technologies. In particular, the well-documented algorithmic bias of AI^41^ and the automation bias of clinicians using decision support tools^42^ that may inadvertently worsen disparities in care, rather than improve them. Furthermore, better rules and security protocols are warranted to safequard data privacy, especially as most AI tools rely on proprietary software owned by technology companies. Lastly, a technocentric approach to care risks flattening patients and clinicians, removing unique characteristics of care^43^, and potentially eroding person-centered care in favor of tech-centered care^44^.

The steering committee urged policy experts to publish guiding principles and governance frameworks for the ethical deployment of digital health tools and AI, ideally co-developed by people with PD, clinicians, and AI experts. Further, governments and health ministries should invest in scalable, regionally appropriate AI tools, leveraging the new technology to supplement, not replace, the human connection. All health care providers, including students, should receive appropriate education in the ethical use of AI in both clinical and research settings.

## Conclusion

With the rising tide of Parkinson’s disease worldwide, countries must come together to develop adequate, appropriate strategies to meet the needs of all people with PD. This change will require attention to all areas of the socio-ecological framework of disease, from the experience of the individual patient to the system-level policies that will provide the help for them to manage their disease. While country-specific strategies may look different, according to local need, global alignment on key priority areas (Table 3) will ensure that mitigation strategies are both equitable and effective.

**Table 3:** Overall Summit Actions and Next Steps

Priority Area	Immediate Actions
**Epidemiology & Economic Impact**	Build registries, improve surveillance, quantify cost of care gaps Simple, actionable campaigns on exercise, toxin exposure, early signs
**Parkinson’s Care Landscape**	Create global care minimum standards, scale caregiver education Launch targeted access pilots in priority countries Train primary care and allied health workers, expand CHW models
**AI & Digital Health**	Develop ethical guidelines, pilot scalable AI tools, engage industry

## Supplementary Information

Supplementary Information

## Data Availability

No datasets were generated or analysed during the current study.
