# Author Correction: Proceedings of the world summit on parkinson’s disease

**DOI:** 10.1038/s41531-025-01189-4

**Published:** 2025-11-21

**Authors:** Sneha Mantri, M. Felice Ghilardi, Nicole Lessard, Monica Norcini, Alessandro Di Rocco, Kristin Wallock, John Lehr, Michael S. Okun

**Affiliations:** 1 Parkinson’s Foundation, New York, NY USA; 2 Duke University School of Medicine, Durham, NC USA; 3 CUNY School of Medicine, New York, NY USA; 4 Fresco Parkinson Institute Italia Onlus, Fiesole, IT Italy; 5 Northwell Health, New York, NY USA; 6 University of Florida, Fixel Institute for Neurological Diseases, Gainesville, FL USA

**Keywords:** Diseases, Health care, Neurology, Neuroscience

Correction to: *npj Parkinson’s Disease* 10.1038/s41531-025-01123-8, published online 14 October 2025

In the original version of this article, there was a typo in the family name of peoples listed in table 1. It was incorrectly written as Felice (Lice) Ghilard and Michael Schwartzchild but should read Felice (Lice) Ghilardi and Michael Schwarzchild. The original article has been corrected.

