# Supplementary material for: Proceedings of the world summit on parkinson’s disease
Source: NPJ Parkinsons Dis. 2025 Oct 14;11:293. doi: 10.1038/s41531-025-01123-8 (PMC12521725; doi:10.1038/s41531-025-01123-8)
Supplement: Supplementary file 1 — Supplementary Information [file 41531_2025_1123_MOESM1_ESM.docx]

| **Participant** | **Affiliation** |
| --- | --- |
| Paolo Calabresi, MD | Fondazione IRCCS Policlinico Gemelli Catholic University |
| Max Coslov, MPhil | Edmund J. Safra Foundation |
| Silke Cresswell, MD | University of British Columbia |
| Alessandro Di Rocco, MD | Northwell Health/ Fresco Parkinson Foundation Institute Italia/Parkinson's Foundation |
| Rachel Dolhun, MD DipABLM | Michael J. Fox Foundation for Parkinson's Research |
| Tarun Dua, MD | World Health Organization |
| Felice (Lice) Ghilardi, MD | The City University of New York |
| Ihtsham Haq, MD FAAN | University of Miami |
| John Lehr, MA | Parkinson's Foundation |
| Nicole Lessard, RN | Parkinson's Foundation |
| Sneha Mantri, MD MS | Duke University/Parkinson's Foundation |
| Danni Manzi | Parkinson's UK |
| Janis Miyasaki, MD MEd FRCPC FAAN | University of Alberta/Parkinson’s Foundation |
| Cathy Molohan, DPhil | Parkinson's Advocate |
| Elena Moro, MD | European Academy of Neurology |
| Monica Norcini, PharmD PhD | Fresco Parkinson Institute Italia |
| Njideka Okubadejo, MBCHB FMCP | University of Lagos |
| Michael S. Okun, MD | University of Florida |
| Eli Pollard, MA | World Parkinson Congress |
| Artur Schuh, MD PhD | Universidade Federal do Rio Grande do Sul |
| Michael Schwarzchild, MD PhD | Massachusetts General Hospital/Harvard University |
| Frederic Seghers, MS | Clinton Health Access Initiative |
| Cholpon Shambetova, MD | Kyrgyz State Medical Academy, University of Luebeck |
| Caroline Tanner, MD PhD | University of California, San Francisco |
| Kristin Wallock, OTD MS OTR/L | Parkinson's Foundation |
| Benjamin Walter, MD MBA | Cleveland Clinic |
